# Therapeutic Targeting of the General RNA Polymerase II Transcription Machinery

**DOI:** 10.3390/ijms21093354

**Published:** 2020-05-09

**Authors:** Ryan D. Martin, Terence E. Hébert, Jason C. Tanny

**Affiliations:** Department of Pharmacology and Therapeutics, McGill University, Montreal, QC H3G 1Y6, Canada; ryan.martin@mail.mcgill.ca

**Keywords:** RNA polymerase II, transcription, CDK inhibitor, CDK7, CDK9, CDK12

## Abstract

Inhibitors targeting the general RNA polymerase II (RNAPII) transcription machinery are candidate therapeutics in cancer and other complex diseases. Here, we review the molecular targets and mechanisms of action of these compounds, framing them within the steps of RNAPII transcription. We discuss the effects of transcription inhibitors in vitro and in cellular models (with an emphasis on cancer), as well as their efficacy in preclinical and clinical studies. We also discuss the rationale for inhibiting broadly acting transcriptional regulators or RNAPII itself in complex diseases.

## 1. Introduction

Changes in the regulation of gene transcription by RNA polymerase II (RNAPII) underlie virtually every complex human disease. Such changes have been most extensively documented in cancer, and gene expression signatures unique to particular forms of cancer are rich resources for identifying routes to novel, targeted therapies [1]. Moreover, such resources and their associated therapeutic potential are also being developed for diabetes, neurological disorders, immune disorders, and cardiovascular disease [2]. RNAPII transcription involves a set of core (“general”) transcription factors that act on all genes transcribed by RNAPII. A much more diverse set of gene-specific and cell-type-specific transcriptional regulators act by binding to specific sites in the genome and engaging in protein–protein interactions with general transcriptional regulators that serve to “recruit” core machinery to a particular site [3,4]. Altered patterns of gene expression in disease tend to reflect changes in a relatively small percentage of genes, and thus efforts to prevent these effects have focused on targeting gene-specific, disease-specific transcriptional regulators. As many of these factors lack enzymatic activity and work through specific protein–DNA and protein–protein interactions, this strategy has proven challenging [5,6]. Surprisingly, targeting general components of the transcription machinery, which are involved in transcription of a plurality of genes, has emerged as a potential alternative strategy. This approach is limited by the broad effects of such therapeutic agents on gene expression. Nonetheless, from a therapeutic perspective, it may be easier to reduce the activity of factors that act generally in transcription and work around the inevitable unwanted effects than to ablate factors that act more specifically. Furthermore, cancer cells often become dependent on abnormally high levels or activity of certain general transcription regulators for their continued proliferation, making these factors attractive targets in their own right [7].

Here we review therapeutic strategies that target general RNAPII transcription regulators. We focus primarily on inhibitors of transcription-associated cyclin-dependent kinases (CDKs), found to be important targets of inhibitors that were originally sought for their effects on cell cycle progression. We also highlight non-CDK targets within the general transcriptional machinery, including RNAPII itself. For each target we discuss its mechanism of action during transcription and evidence for clinical efficacy of its cognate inhibitor, with a view toward understanding how balance is struck between therapeutic benefit and toxicity.

## 2. A CDK-Centered Overview of RNAPII Transcriptional Regulation

RNAPII transcription occurs in three steps: initiation, elongation, and termination. Initiation involves the recognition of core promoter elements on DNA by RNAPII and general transcription factors and synthesis of the first few nucleotides of the RNA transcript. The steps in initiation were defined biochemically using purified factors and order-of-addition experiments, but larger assemblies of multiple factors may in fact engage promoters in vivo [8]. A key step in initiation is promoter binding by TFIID, a multi-subunit factor containing TATA-binding protein (TBP) and TBP-associated factors (TAFs). Canonical promoters contain a TATA box (consensus sequence TATA(A/T)A(A/T)(A/G)) around 30 base pairs upstream of the transcription start site [8]. However, TFIID binding, through additional core promoter elements recognized by TAFs, is also essential for transcription at non-TATA promoter sites [9]. The TBP-promoter complex is stabilized by subsequent binding of TFIIA and TFIIB, with TFIIB playing a key role in subsequent recruitment and positioning of RNAPII. The preinitiation form of RNAPII is thought to bind the promoter in a complex with TFIIF, which stabilizes the complex of RNAPII with the promoter and other initiation factors and helps to ensure that RNAPII initiates transcription at the appropriate location [3,8].

Before RNA synthesis commences, RNAPII is also bound by Mediator, a 23-subunit assembly that further stabilizes the initiating RNAPII complex and acts as a physical link to gene-specific transcriptional activators bound to regulatory sites on DNA. Mediator is a dynamic complex composed of head, middle, tail, and kinase modules [10]. The head and middle modules are essential for cell viability and global RNAPII transcription, and structural studies demonstrate that these modules make key contacts with the initiating RNAPII complex. In contrast, the tail and kinase modules are not essential but are important for the function of specific transcriptional activators. The activation domains of a number of transcription factors, including sterol receptor element binding protein 1 (SREBP-1), E1A (an adenoviral activator protein), and hepatic nuclear factor alpha (HNFα), have been shown to directly contact the tail, while tail-activator interactions are functionally important for transcriptional activation [11]. 

**Figure 1:**
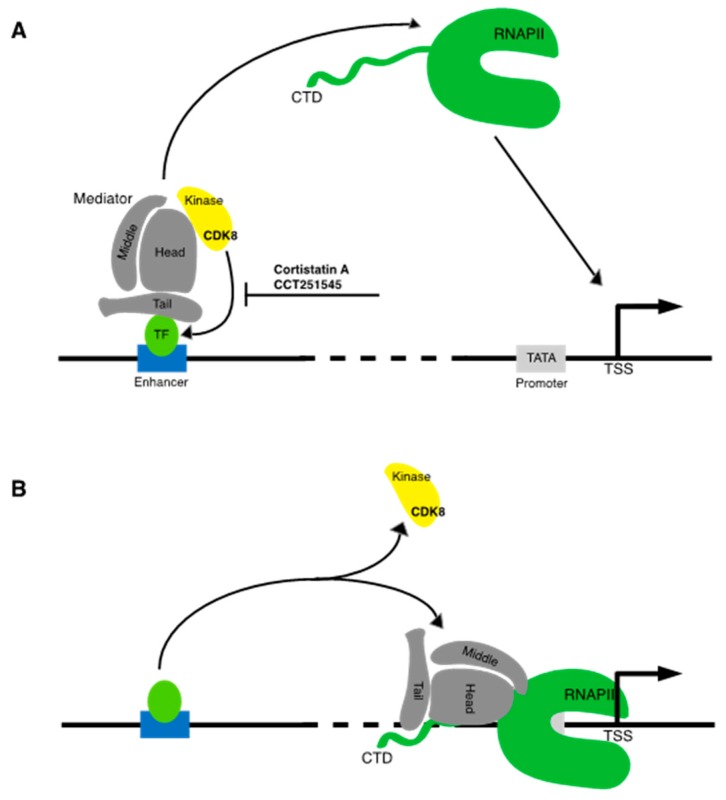
Modulation of gene-specific transcription factor function by Mediator-associated kinase CDK8. (**A**) Transient interaction of Mediator with a transcription factor (TF) bound at an enhancer allows modulation of transcription factor function by CDK8, perhaps through direct phosphorylation. CDK8 inhibitors discussed in the text are indicated. “Head,” “middle,” “tail,” and “kinase” refer to modules of the Mediator complex. CTD = RPB1 C-terminal domain; TSS = transcription start site. The dashed line indicates the variable distance between enhancer and promoter sequences. General transcription factors bound to the promoter with RNA polymerase II (RNAPII) are omitted for clarity. (**B**) Following interaction with gene-specific transcription factors, Mediator associates with RNAPII at the core promoter site to activate transcription. The kinase module is released.

The kinase module does not directly interact with activators, but instead seems to regulate a dynamic transition between Mediator bound at enhancer elements (DNA elements that comprise specific binding sites for DNA-binding transcriptional activators), and Mediator bound at the core promoter [12] (Figure 1A). The kinase module is so named as it contains CDK8, a member of the transcription-associated subclass of CDKs, and its cognate cyclin CCNC. The Mediator subunits MED12 and MED13 serve to link this kinase–cyclin pair to the rest of Mediator. A variant of the kinase module containing CDK19, a CDK8 paralog, has also been identified [10]. Biochemical and structural studies demonstrate that association of the kinase module with Mediator prevents Mediator–RNAPII interaction [13]. The form of Mediator containing this module is thought to act specifically at enhancer sites in a transient manner, such that kinase module is ejected as Mediator engages RNAPII at core promoter sites (Figure 1B) [12,14,15]. How the kinase module regulates transcription factor function is poorly understood; there is evidence that it can play a positive or a negative role. CDK8 directly phosphorylates several transcription factors, including E2F and SMADs, thereby enhancing their activity [10].

Formation of the Mediator-RNAPII complex requires an interaction between the Mediator “head” module and the C-terminal domain (CTD) of RPB1, the largest RNAPII subunit [16]. The CTD is unstructured and composed of multiple repeats of a 7-residue motif (YSTPSPS) [17]. CTD length appears to have co-evolved with genome complexity in eukaryotes; the budding yeast CTD contains 26 heptad repeats, whereas the human CTD contains 52. The CTD is important for all stages of RNAPII transcription and serves as a binding site for numerous regulators in addition to Mediator, including diverse mRNA processing factors and histone-modifying enzymes. CTD functions at different phases of transcription are governed by phosphorylation of specific residues within the heptad repeat sequence. Mediator engages the CTD when it is in a hypophosphorylated form, which is characteristic of the preinitiation complex.

Unwinding of promoter DNA and initial RNA synthesis requires the engagement of the TFIIE and TFIIH complexes. TFIIH is a 10-subunit complex that includes ATP-dependent helicase subunits (XPB and XPD) and a kinase module composed of another transcription-associated CDK family member CDK7, its cognate cyclin CCNH, and MAT1 [3]. Promoter DNA opening by TFIIH proceeds via an unconventional activity of the XPB helicase, which serves to translocate DNA ahead of the polymerase into the active site. This creates torsional strain, leading to melting of a 14 bp segment surrounding the promoter to form a transcription “bubble” on which RNA synthesis begins [18]. TFIIH helicases are also important for transcription-coupled DNA repair, and mutations in XPB and XPD are associated with the human diseases xeroderma pigmentosum, tricothiodystrophy, and Cockayne’s syndrome. 

**Figure 2:**
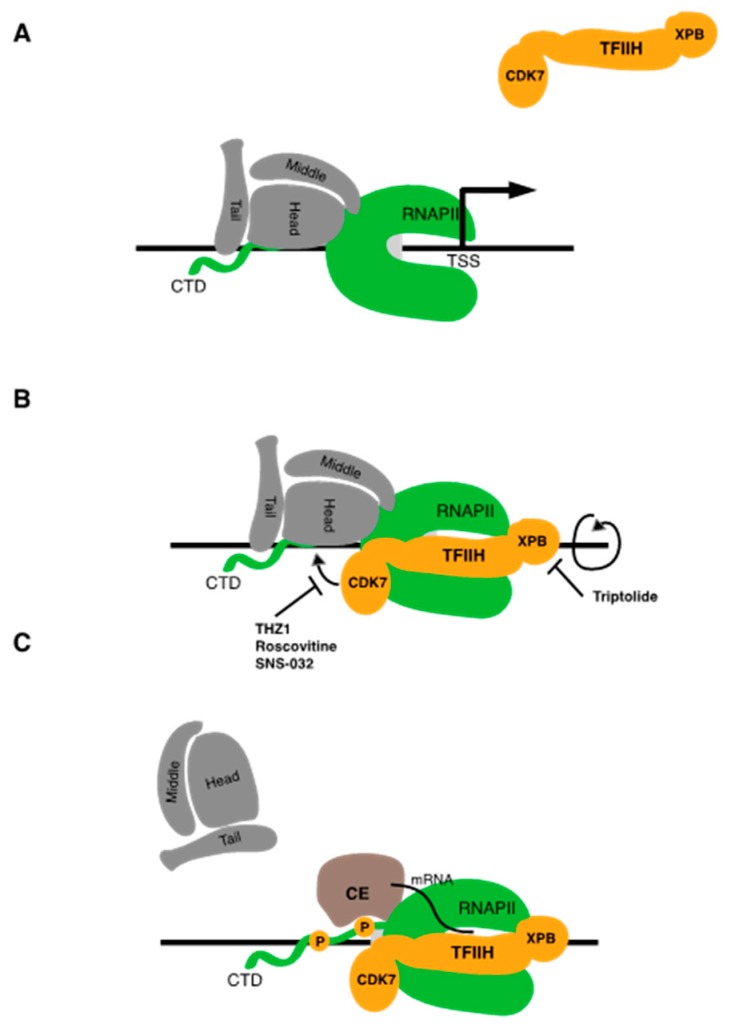
Inhibition of TFIIH blocks RNAPII transcription initiation. (**A**) TFIIH associates with the RNAPII–Mediator complex bound at the core promoter. (**B**,**C**) ATP-dependent DNA translocase activity of XPB leads to unwinding of promoter DNA, while phosphorylation of CTD serine 5 residues (orange circles) leads to dissociation of Mediator and binding of the mRNA capping enzymes (CE). Inhibitors of TFIIH-associated activities discussed in the text are indicated.

Upon initiation, CDK7 phosphorylates the RNAPII CTD at the serine 5 position of the heptad repeat. This serves a dual purpose—it decreases the affinity of Mediator for RNAPII, resulting in its removal from the transcription complex, and it creates a binding site for enzymes involved in the addition of the methyl-guanosine cap structure to the 5′ end of the nascent mRNA transcript emerging from the RNAPII active site [16,19] (Figure 2A). CDK7 also phosphorylates TFIIE, a factor that stimulates ATPase and kinase activities of TFIIH within the initiation complex. TFIIE phosphorylation disrupts its interaction with RNAPII, thereby allowing the binding of the elongation factor Spt5 (discussed in more detail below) [20]. Thus, CDK7 activity is critical for driving the transition from initiation to elongation. CDK7 also has a second, transcription-independent function as a CDK-activating kinase (CAK). CDK activation occurs through phosphorylation of the “T-loop” in the kinase active site [21,22]. Thus, CDK7 acts directly in both regulation of RNAPII transcription and cell cycle progression.

**Figure 3:**
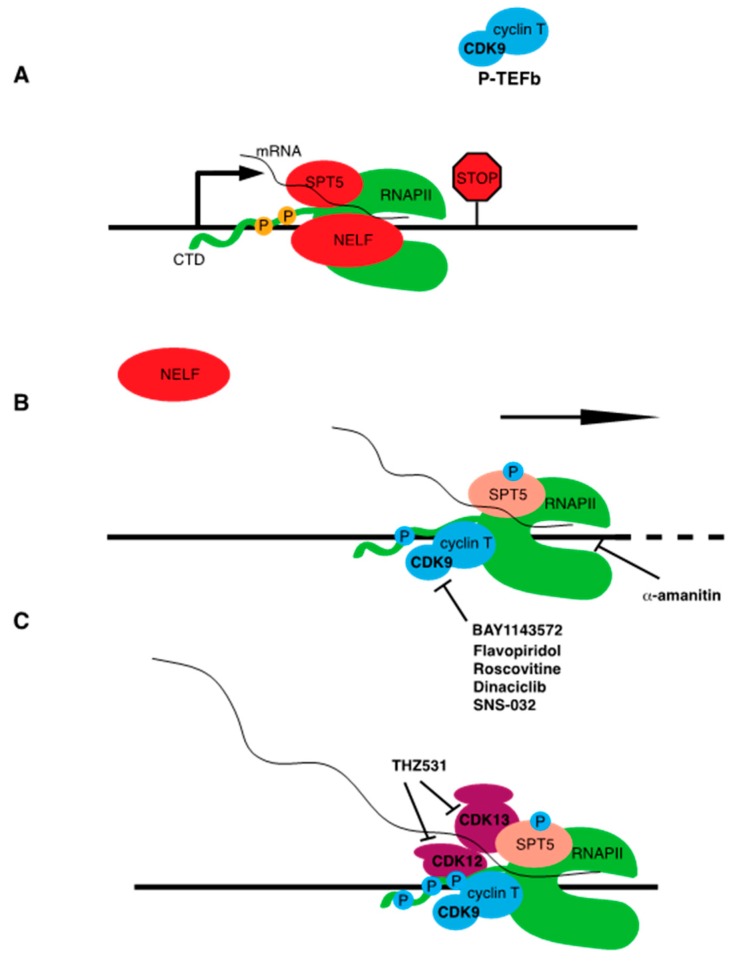
Inhibition of RNAPII transcription elongation. (**A**) Promoter-proximal pausing of RNAPII is enforced by SPT5 and the NELF complex. (**B**) P-TEFb engages the paused polymerase and phosphorylates SPT5 (blue circle), the RPB1 CTD (on multiple sites including serine 2, shown as blue circle), and NELF-E, leading to NELF dissociation and pause release. Inhibitors of CDK9 discussed in the text are indicated. Elongation of the nascent mRNA chain is inhibited by α-amanitin. (**C**) CDK12 CDK13 promote CTD serine 2 phosphorylation, mRNA processing, and elongation.

At many gene sites, elongation of RNAPII becomes paused ~50 nucleotides downstream of the transcription start site. Pausing allows for rapid and synchronous activation of transcription upon pause release, a regulatory feature shown to be critical for certain classes of genes such as those involved in stress responses or in embryonic development [23,24]. Pausing may also function as an mRNA quality control “checkpoint,” ensuring that RNAPII elongation is properly coupled to mRNA processing. SPT5 is required to establish pausing, along with the negative elongation factor complex NELF. These factors directly bind to the early elongation complex. A preformed complex of transcribed RNAPII and SPT5, including the nascent RNA, are prerequisites for stable NELF association [25] (Figure 3). Release of RNAPII from the paused state is mediated by CDK9 and the cyclin CCNT. This CDK-cyclin pair is known as positive transcription elongation factor b (P-TEFb), a complex that is biochemically defined to stimulate the release of RNAPII from pause sites on DNA templates in vitro [26]. P-TEFb directly phosphorylates SPT5 to effect pause release. SPT5, as with RPB1, contains a C-terminal region (CTR) that is composed of a repeated amino acid motif [27]. The CTR repeat motif is more degenerate and less conserved than the CTD on RPB1 (the consensus sequence in humans is TPxxLxx), however direct phosphorylation of SPT5 CTR residues by CDK9 appears to be a broadly conserved transcriptional regulatory mechanism. CDK9 activity also promotes phosphorylation of the NELF-E subunit of NELF. Upon release from the paused state, NELF disengages from the elongation complex and Spt5 is converted from a negative to a positive regulator of elongation [23]. Whereas CDK7 can be viewed as triggering the initial stages of RNAPII elongation, CDK9 creates a fully productive RNAPII elongation complex that is able to engage mRNA splicing and termination factors, as well as enzymes that co-transcriptionally modify the structure of chromatin.

Recent studies have highlighted the importance of liquid–liquid phase separation (LLPS) in mediating some of the key events in the RNAPII transcription process. LLPS typically involves heterogeneous interactions between structurally disordered protein domains, forming large assemblies that behave as distinct compartments or “phases” [28]. The RPB1 CTD is one such intrinsically disordered domain, and LLPS has been shown to govern its interactions with regulators at different stages of transcription. For example, assemblies involving Mediator and the unphosphorylated form of the RPB1 CTD, as well as those involving phosphorylated CTD and mRNA splicing factors, both occur in the context of phase-separated compartments. Intriguingly, CTD phosphorylation may drive its transition through distinct compartments in concert with the transition from initiation to elongation [29]. Thus, CDK7 and CDK9 not only alter phospho-specific protein interactions with RNAPII complexes but may also facilitate phase transitions and higher order assemblies involving additional factors.

In vivo, P-TEFb promotes phosphorylation of the RNAPII CTD at the serine 2 position in the heptad repeat. Serine 2 phosphorylation is a conserved mark of elongating RNAPII, is enriched near the 3′ ends of gene coding regions, and serves as a binding site for mRNA termination factors [17]. CDK9 phosphorylates the CTD directly, but it is likely that its role in serine 2 phosphorylation is at least in part indirect, stemming from its requirement for RNAPII to efficiently elongate to the 3′ ends of genes. Two other CDKs, CDK12 and CDK13 (which form separate complexes with the cyclin CCNK), also contribute directly to serine 2 phosphorylation in vivo [30,31] (Figure 3). Both kinases are also required for normal premRNA splicing patterns and termination, although these functions likely depend in part on direct interactions with RNA processing factors [32]. Although serine 2 phosphorylation is a universal marker of RNAPII elongation, CDK12 and CDK13 are individually implicated in expression of specific subsets of genes involved in the DNA damage response and in protein translation. This is likely a reflection of the contribution of multiple kinases to Rpb1 serine 2 phosphorylation patterns in vivo. Further research is needed to determine the significance of these gene-specific effects and whether they could indicate a gene-specific function for serine 2 phosphorylation. CDK12 is unique among transcription-associated CDKs in that it is subject to somatic genetic alterations in cancer. Inactivating mutations in CDK12 are found in ovarian carcinoma, suggesting that it can act as a tumor suppressor [33].

## 3. Catalytic Mechanism of RNAPII Transcription

The previous section highlights CDKs as enzymes with multiple general roles in RNAPII regulation. The catalytic mechanism for RNA chain elongation by RNAPII is also a potential therapeutic target. X-ray crystallography studies have provided deep insight into these steps, which appear to be fundamental to DNA-dependent RNA polymerization reactions in all three branches of life. The RNAPII active site is comprised of structural elements formed by the large RNAPII subunits Rpb1 and Rpb2 [34]. Catalysis begins with selection of the appropriate nucleotide triphosphate (NTP) substrate. Upon entering the active site, an NTP base pairs with the DNA template at just 3′ of the growing RNA chain (the 3′ 9 nucleotides of which are hybridized to the template DNA strand abutting the active site). Initially, this interaction occurs in the context of an open, catalytically inactive conformation of the active site, however recognition of the cognate NTP induces active site closure. This involves a switch in the conformation of the “trigger loop” from a mobile, unstructured form to an alpha-helical hairpin. This closes the active site and establishes the contacts needed for catalysis [34,35]. 

Catalysis proceeds via a two-metal ion mechanism, in which one Mg^2+^ ion binds the 3′ end of the RNA and a second Mg^2+^ ion binds the NTP [36]. This facilitates nucleophilic attack of the NTP α-phosphate by the RNA 3′-OH group, leading to nucleotide addition, release of pyrophosphate, and unfolding of the trigger loop. This leaves the newly incorporated nucleotide in the RNAPII active site. In order for the nucleotide addition cycle to restart, RNAPII must translocate along the DNA template to displace the growing RNA and allow the next NTP to enter the active site. Crystallographic and biochemical evidence suggest that unfolding of the trigger loop after catalysis, as well as movement of the adjacent “bridge helix” that abuts the 3′ end of the RNA chain, translocate the new base pair into a “pretemplating” position above the bridge helix. Re-setting of the bridge helix to its original position moves the template DNA strand into position for a new round of nucleotide addition [34,37].

## 4. CDK Inhibitors Affecting RNAPII Transcription

The first generation CDK inhibitors were discovered and characterized in the 1990s in an effort to target cell proliferation in cancer. These were relatively non-specific and can be thought of as “pan-CDK” inhibitors. We focus on flavopiridol, roscovitine, SNS-032, and dinaciclib, compounds in this class with well-defined effects on transcription. We note that these represent only a subset of clinically relevant compounds in this class (see Table 1 for other examples) [38,39], although their clinical utility has been limited by their toxicity. Recently inhibitors that are more selective for individual CDKs have been developed. These include some compounds that are now widely used clinically, notably CDK4 and CDK6 inhibitors used to treat estrogen-receptor positive breast cancer [40]. We will focus on compounds that target the transcription-associated class of CDKs: THZ1 for CDK7, cortistatin A for CDK8, BAY 1143572 for CDK9, and THZ531 for CDK12 and CDK13 (Table 1). Below, we discuss initial research demonstrating the effects of these agents on general RNAPII transcription and their potential translation into therapeutic agents. Where applicable, we also review how these inhibitors have been used in clinical settings, primarily in the context of cancer (also see Table 1). 

### 4.1. Pan-CDK Inhibitors

#### 4.1.1. Flavopiridol

Flavopiridol is a semi-synthetic flavonoid derived from the chromone alkaloid rohitukine. An initial study hinted at its therapeutic potential by demonstrating flavopiridol’s ability to inhibit proliferation of two different breast cancer cell lines [41]. Subsequently, flavopiridol was found to be not only cytostatic but cytotoxic to a range of human cancer cell lines, including a non-dividing cell line [42]. In the years that followed, the toxicity of flavopiridol was demonstrated in a variety of cancer cell lines and several in vivo animal models [53]. As such, it was the first pan-CDK inhibitor to enter clinical trials in 1998 for refractory neoplasms [54], with over 60 trials completed between 1998 and 2014 [55]. 

Flavopiridol is a potent ATP-competitive inhibitor of CDK1, CDK2, CDK4, CDK6, and CDK9, for which its IC_50_ values range between 20 and 60 nM; it has less activity against CDK7 (IC_50_ = 875 nM) [56,57]. Flavopiridol has off-target effects on kinases outside of the cyclin-dependent kinase family, having the most potent effects on glycogen synthase kinase-3 (IC_50_ = 280 nM) [58]. Although its main cellular effects were initially attributed to inhibition of the cell cycle kinases, there were early indications of an additional mechanism. For example, flavopiridol caused decreased cyclin D expression, as well as decreased CDK2 and CDK4 activity, in breast cancer cell lines [59]. Further hints at a transcriptional effect were observed in a human B cell leukemia cell line, in which flavopiridol treatment lead to the downregulation of the antiapoptotic Bcl-2 transcript, an effect not observed in response to flavonoids with no CDK inhibitory action [60]. A direct effect of flavopiridol on transcription was first demonstrated using a reporter gene under the control of the cyclin D1 promoter [61], marking the beginning of a shift in our understanding of how flavopiridol exerted its cellular effects. 

Insight into the effect of flavopiridol on RNAPII-mediated transcription came from the finding that it behaved similarly to 5,6-dichloro-1-β-D-ribofuranosylbenzimidazole (DRB), a CDK9-selective inhibitor, in an in vitro transcription assay. Flavopiridol inhibited CDK9 with a K_i_ of 3 nM, whereas its Ki for other CDKs was in the range of 20–60 nM [62]. Further work by Chao et al. demonstrated an effect of flavopiridol on transcription through RNAPII in cells using nuclear run-on assays [63]. Similar to DRB, flavopiridol was found to have global effects on transcription [64]. Unstable mRNAs, including those encoding key cell cycle regulators and Bcl-2, were among the most sensitive, in agreement with the observed cytostatic and cytotoxic effects of this compound. The authors proposed that because of this transcriptional effect, flavopiridol may exert specific activity on cancers that are dependent on unstable mRNAs, such as the antiapoptotic Bcl-2 family. Indeed, flavopiridol blocks the growth of both multiple myeloma and chronic lymphocytic leukemia (CLL) cells, in which downregulation of Mcl-1, a Bcl-2 family member, was found to be flavopiridol’s primary mechanism of action. 

Flavopiridol (alvocidib) has entered several clinical trials since 1998 for a variety of different cancer types. Phase II trials for various solid tumors and mantle cell lymphoma with 72 h continuous infusion showed no significant clinical response, even though flavopiridol plasma concentrations reached concentrations required in vitro for CDK inhibition [65,66,67,68,69]. A modest response was observed in a phase II trial of mantle cell lymphoma [43]. The low efficacy of flavopiridol in clinical studies compared to its in vitro activity may have been due to its tight binding to human plasma proteins. Another trial was initiated with a pharmacologically derived schedule to achieve higher plasma concentrations in patients with refractory, genetically high-risk CLL [44,70]. A phase II trial based on these results demonstrated significant single molecule clinical activity in patients with high-risk genetic features, with an overall response rate of between 30% and 40% [71,72].

#### 4.1.2. Roscovitine (Seliciclib) 

Roscovitine was discovered in 1997 as an inhibitor of meiosis in starfish oocytes [73]. It was subsequently found to be a potent CDK inhibitor; it inhibits CDK1, CDK2, CDK5, CDK6, CDK7, and CDK9, with IC_50_ values below 1 μM [73,74,75]. Similarly to flavopiridol, roscovitine targets the ATP binding sites on CDKs. Roscovitine was shown to be cytotoxic in a panel of human cancer cell lines with an average IC_50_ of 15.2 μM [76]. Unlike flavopiridol, which causes global down regulation of gene expression, roscovitine led to a unique gene expression profile with a smaller subset of sensitive genes [64]. Genes responsive to roscovitine are crucial for the induction of apoptosis in a variety of cancers, and include Bcl-2 [77], survivin [78], and the p53-upregulated modulator of apoptosis (PUMA) [79]. Roscovitine is thought to directly block transcription of some genes by inhibiting CDK7 and CDK9. 

In an early in vivo study, roscovitine reduced the formation of tumors from human colorectal cancer xenografts by up to 62% [76]. Subsequent studies demonstrated roscovitine’s efficacy, utilizing xenograft models of various cancers [45,80]. Although roscovitine was efficacious with a variety of administration methods, pharmacodynamic and pharmacokinetic analysis with mice determined oral administration to be the optimal method of delivery [80]. Clinical trials have thus far failed to show any efficacy for roscovitine as a monotherapy for cancer [55,81]. However, there is mounting evidence that roscovitine has synergistic effects with other anticancer agents and may serve best as an adjunct therapy to current treatments [82]. Roscovitine may also be an important chemical scaffold for development of CDK inhibitors with improved potency and specificity [83].

#### 4.1.3. SNS-032 

SNS-032 was developed by Bristol–Myers Squibb as a CDK2 Inhibitor. X-ray crystallography studies demonstrated that SNS-032 competes with ATP for binding to CDK2 [46] and inhibits CDK2, with an IC_50_ of 48 nM. However, it was subsequently found to inhibit CDK7 and CDK9 with similar potency (IC_50_ = 4 nM and 62 nM, respectively) [84]. In support of its effects on global RNAPII transcription, SNS-032 treatment led to a decrease in phosphorylated Ser2 and Ser5 of RNAPII CTD and decreased expression of antiapoptotic proteins Mcl-1 and XIAP in a variety of cancers, such as multiple myeloma [84], acute myeloid leukemia (AML) [85], neuroblastoma [86] and breast cancer [47]. These effects were also observed in a phase I clinical trial with patients with chronic lymphocytic leukemia and multiple myeloma [87]. Relative to flavopiridol, this compound had favorable pharmacokinetic and pharmacodynamic properties and showed better efficacy in preclinical studies [46], although no further clinical development has been announced.

#### 4.1.4. Dinaciclib 

Dinaciclib was discovered in a screen to identify CDK1 and CDK2 inhibitors beginning with a pyrazolo[1,5-a]pyrimidine scaffold [88]. Hit compounds were screened in a combination of assays, which included an in vivo screen that assessed the efficacy versus tolerability profiles in the A2780 ovarian carcinoma xenograft model. Further characterization revealed that dinaciclib potently inhibits CDK1, CDK2, CDK5, and CDK9, with IC_50_ values of 3, 1, 5, and 9 nM, respectively. Similar to other pan-CDK inhibitors, dinaciclib induced apoptosis and decreased expression of antiapoptotic genes such as Mcl-1 and survivin, hallmarks of impaired CDK7 and CDK9 function [89]. Dinaciclib has several properties that make it an attractive potential therapeutic. Compared to flavopiridol, dinaciclib was more selective for the CDK family over other kinases. Dinaciclib also maintains its efficacy in vivo in animal models despite the fact that it is rapidly cleared from plasma, in contrast to other pan-CDK inhibitors [90]. A recent phase III trial for relapsed and refractory chronic lymphocytic leukemia showed considerable promise, with an overall response rate of 40% (42 patients) and an acceptable safety and tolerability profile [91]. Other clinical trials with dinaciclib are currently underway.

### 4.2. Selective CDK Inhibitors

#### 4.2.1. THZ1

THZ1 was identified through cell-based and kinase selectivity profiling of a library of inhibitors targeting the ATP-binding site of CDK7 [92]. THZ1 is a unique CDK inhibitor, in that it acts covalently and irreversibly at a site outside of the catalytic domain. A cysteine-reactive acrylamide moiety on THZ1 forms a covalent bond with C312 of CDK7, which extends in front of the kinase domain and places THZ1 in close proximity to the ATP binding site, as determined by crystallography. CDK7 is inhibited with an IC_50_ of 3.2 nM, and the unique mechanism results in remarkable selectivity, as the IC_50_s for other CDKs are in the micromolar range [92]. CDK12, which contains a similarly localized cysteine residue, is the lone exception, although its IC_50_ is almost 10-fold higher compared to CDK7. Preclinical studies have demonstrated the effectiveness of THZ1 to prevent cell proliferation and induce apoptosis in a variety of cancer types, such as high grade gliomas [93], T-cell acute lymphoblastic leukemia (T-ALL) [92], MYCN-dependent neuroblastoma [94], small-cell lung cancer [95], triple negative breast cancer [96,97], peripheral T-cell lymphomas [98], and esophageal squamous cell carcinoma [99]. Importantly, overexpression of the CDK7 C312S mutant renders cancer cell lines resistant to many of the effects of THZ1, confirming its specificity for CDK7 in cells [94]. Transition to in vivo mouse studies demonstrated limited systemic toxicity and significant reduction in tumor size; further preclinical and clinical characterization is ongoing [92]. Optimization of THZ1 pharmacokinetic properties has also led to the development of the next-generation covalent inhibitor SY-1365 [100]. A phase I study is currently being completed in patients with advance solid tumors, ovarian cancer, and breast cancer.

THZ1 treatment led to a dose-dependent decrease in RNAPII CTD phosphorylation at Ser2, Ser5, and Ser7 sites in multiple cancer cell lines [48,92,93,94,95,96,98,99]. This reflects decreased activity towards direct targets of CDK7 on the CTD (Ser5, Ser7), as well as an indirect effect on Ser2 due to the role of CDK7 as a CAK for CDK9 [20]. In T-ALL cells, THZ1 affected CAK activity (as measured by reduction in T-loop phosphorylation on CDK1, CDK2, and CDK9) to a lesser extent than RNAPII CTD phosphorylation [92]. Thus, proliferation arrest by THZ1 is likely predominantly due to effects on transcription rather than reduced activation of the CDKs [94,97].

Owing to the fact that THZ1 specifically targets RNAPII transcription, it has been a useful tool for understanding the importance of general transcription mechanisms for sustaining cancer cell proliferation. Sensitivity to THZ1 in cancer cell lines correlates with the extent to which oncogenic, gene-specific transcription factors are overexpressed (as assessed by gene ontology term enrichment analysis of overexpressed genes) [94,95,99]. A striking example of this was demonstrated in studies of neuroblastoma cell lines, in which sensitivity to THZ1 was almost 10-fold greater in cells harboring amplified levels of the MYCN transcription factor compared to those without amplified MYCN [94]. Such selective sensitivity to impairment of general transcriptional regulators is observed in a variety of cancers and has been termed “transcriptional addiction” [7]. This phenomenon arises from dramatically enhanced expression or activity of gene-specific transcription factors rather than any alteration of general regulators themselves. Overly active gene-specific transcription factors are frequently found to act at super-enhancer sites, clusters of transcription factor binding sites that typically comprise the DNA regulatory elements controlling expression of master developmental regulators [49]. Super-enhancers and the genes they control account for a large proportion of transcriptional defects in “addicted” cancers. Concordantly, super-enhancer-linked genes were disproportionately affected by THZ1 in a variety of cancers [92,95,96,97,99]. The extent to which super enhancer activity may predict response to other inhibitors of RNAPII transcription in diseases other than cancer is an important question for further study.

#### 4.2.2. Non-Covalent CDK7 Inhibitors

BS-181 was derived by computational modelling, beginning with core fragments of roscovitine and subsequent docking studies. These studies identified a molecule favorable to the CDK7 active site over CDK2, CDK5, and CDK6 based on docking scores. In an in vitro kinase assay, BS-181 inhibited CDK7 with an IC_50_ of 21 nM, demonstrating 25-fold greater potency than roscovitine (IC_50_ = 510 nM). BS-181 inhibited all other tested CDKs with IC_50_ values of 880 nM or greater [101]. Furthermore, BS-181 more effectively inhibited Ser5 phosphorylation on the RNAPII CTD than roscovitine. BS-181 inhibited cell proliferation in a panel of cancer cell lines and growth of tumor explants in nude mice. Although BS-181 was able to reduce tumor growth in vitro and in vivo, it was found to be 500 times less potent than THZ1 in triple negative breast cancer [96]. Through optimization of the BS-181 structure, the Cdk7 competitive inhibitor ICEC0942 (Cdk7 IC_50_ = 40 nM) was developed [102], for which phase I clinical studies are underway.

#### 4.2.3. Cortistatin A

Cortistatin A is one of eleven natural steroidal alkaloids in the cortistatin family that have been isolated from *Corticium simplex* [103,104]. The initial characterization demonstrated potent antiproliferative effects (IC_50_ = 1.8 nM) and reduced VEGF-induced migration of umbilical vein endothelial cells (HUVECs), without having an effect on phosphorylation of ERK1/2 and p38. Further insight into the mechanism of action for these compounds was gained via a high throughput screen with a panel of 402 kinases that revealed ROCKI and II, as well as the Mediator-associated kinases CDK8 and CDK19 [105]. Quantitative affinity measurements indicated that CDK8 and CDK19 were the preferred targets, with K_d_s values of 17 and 10 nM, respectively, compared to >200 nM for ROCKI and ROCKII. Cortistatin A is selective for CDK8 and CDK19 due to remarkable shape complementarity with the ATP binding site. Crystallogaphy studies implicated a tryptophan residue in the ATP binding pocket unique to CDK8 and CDK19 in cation–π interactions with the dimethylamine group of cortistatin A [106].

Both in vitro and in vivo mouse models of acute myeloid leukemia were used to demonstrate the antiproliferative activity of cortistatin A [50,106]. For example, once daily intraperitoneal injection of 0.16 mg kg^-1^ of cortistatin A led to a 71% decrease in tumor volume in a SET-2 acute myeloid leukemia (AML) xenograft mouse model. Surprisingly, suppression of AML growth was associated with increased expression of super-enhancer-linked genes. The mechanism for this repressive effect of CDK8/19 seems to involve phosphorylation of the transcription factor STAT1, which is prevented by cortistatin A [50]. These studies demonstrate that cortistatin A is a promising cancer therapeutic and will be advanced by ongoing preclinical research. They also suggest that cancer cells need to maintain an optimal level of expression of super-enhancer-linked genes for sustained proliferation. This implies that a more nuanced formulation of the “transcriptional addiction” concept, which does not solely invoke increased transcriptional activity, should be considered.

#### 4.2.4. Other Mediator Kinase Inhibitors

Links between Mediator kinase activity and STAT1 function in cancer have been strengthened by the study of two other inhibitors, CCT251545 [107] and SEL120-34A [108]. Both potently and selectively inhibit CDK8 and CDK19 (IC_50_ in the 5–10 nM range). The co-crystal structure of CCT251545 bound to CDK8/cyclin C revealed that a loop region in the C-terminal domain of CDK8, far-removed from the kinase domain itself, folds over the active site and forms a hydrogen bond with the inhibitor. This unique binding mode likely contributes to the CDK8 specificity of CCT251545 [107]. This loop is also in proximity to the active site in the structure with cortistatin A [106]. Gene expression analysis in LS174T and COLO205 colon carcinoma cell lines demonstrated selective modulation of genes regulated by STAT signalling. Furthermore, CCT251545 inhibited growth of Wnt-driven breast and colorectal cancer cells in xenograft models [107]. However, in vivo studies have indicated significant toxicity [51]. The dependence of STAT signalling on CDK8 was also found with the specific inhibitor SEL120-34A. Acute myeloid leukemias with elevated phosphorylation of STAT transactivation domains displayed increased sensitivity to SEL120-34A treatment [108].

#### 4.2.5. CDK9 Inhibitors

Whereas recently developed inhibitors of CDK7 and Mediator kinases derive their selectivity from amino acid residues unique to these kinases, selective CDK9 inhibitors recognize subtle structural features of the conserved ATP-binding pocket. As such, these inhibitors tend to retain significant affinity for other kinases, a likely explanation for their limited utility in preclinical and clinical studies [100]. X-ray crystallography studies have compared the binding of DRB, a selective CDK9 inhibitor often used as an experimental tool compound, to complexes of CDK9/cyclin T or CDK2/cyclin A [109]. CDK9 selectivity was associated with (1) stronger halogen bonding between the inhibitor and the kinase hinge region and (2) conformational changes that allowed a greater number of van der Waals contacts with the inhibitor. The theme of conformational flexibility, resulting in effective malleability of the ATP-binding pocket in CDK9, was also noted in subsequent studies of substituted pyrimidine analogs that are selective for CDK9 [52,110]. Remarkably, these compounds made no specific polar contacts with CDK9 as compared to CDK2, and selectivity was imparted entirely by CDK9-specific, inhibitor-induced conformational changes.

Although these and more recently developed CDK9 inhibitors have potent effects on cancer cells in vitro, there is as yet no data on their in vivo efficacy [111,112]. A potential route to clinical development for a CDK9-specific inhibitor is exemplified by BAY 1143572 [113]. This compound is characterized by a benzyl sulfoximine group, which is unique among CDK9 inhibitors. BAY 1143572 is highly potent and specific, with IC_50_ of 6 nM for CDK9 and >470 nM for CDK1, CDK2, CDK3, CDK5, CDK6, and CDK7. In vivo studies with mice xenograft models of human acute myeloid leukemia demonstrated suitable pharmacokinetics and efficacy in reducing tumor growth. In primary adult T-cell leukemia and lymphoma, BAY 1143572 decreased RNAPII phosphorylation and expression of Mcl-1 and Myc [114]. Clinical development of this compound (including a second-generation variant) is currently underway (Table 1).

#### 4.2.6. THZ531

CDK12 and CDK13 crystal structures revealed a surface-exposed cysteine residue at a location corresponding to that of the THZ1 target cysteine in CDK7. Given the known roles of CDK12 in cancer, THZ1 was used as a starting point to develop THZ531, a selective CDK12/13 inhibitor [31]. In vitro kinase assays demonstrated the specificity of THZ531 for CDK12 and CDK13 (IC_50_ = 158 and 69 nM, respectively) over CDK7 and CDK9 (IC_50_ = 8.5 and 10.5 μM, respectively). THZ531 exhibited dose- and time-dependent effects on proliferation and apoptosis in Jurkat T cell acute lymphoblastic leukemia cells. RNA-seq and ChIP-seq for RNAPII phosphorylated at serine 2 revealed that at low doses of THZ531, a small subset of genes involved in the DNA damage response displayed reduced expression. At higher doses sufficient to induce apoptosis, there was a global reduction in serine 2 phosphorylation at gene 3′ ends, reduced RNAPII processivity, and a global reduction in gene expression. The most highly affected genes encoded transcription factors associated with super enhancers and drivers of T-ALL growth, such as RUNX1, TAL1, and GATA3 [31]. Thus, CDK12/13 inhibition may allow the opportunity to finely tune treatment depending on whether the target cells are sensitive to impairment of the DNA damage response or are more generally “transcriptionally addicted.” Its gene-specific effects at low doses could also be exploited in combination with other compounds that target the DNA damage response [100].

## 5. Triptolide Targets TFIIH Subunit XPB

Triptolide is a natural product that is purified from *Tripterygium wilfordii* Hook F, a medicinal plant that is also known as thunder god vine and that has been used in traditional Chinese medicine for centuries [115]. It is a diterpene triepoxide compound with antiproliferative and immunosuppressive activities in vitro and in animal models. Its antiproliferative effect is quite general, as it inhibits growth of all 60 U.S. National Cancer Institute cell lines at nanomolar concentrations. Although several early studies showed that triptolide has general effects on RNA synthesis and the function of a variety of transcription factors in cells, detailed insight into its mechanism of action came more recently upon identification of the TFIIH subunit XPB as a direct binding target [116]. In vitro transcription experiments demonstrated strong selectivity for RNAPII over RNAPI and RNAPIII, and an RNAPII transcription system reconstituted from purified components was used to identify the specific target within the RNAPII general machinery. A key finding in precisely identifying the target was that triptolide effectively inhibited not only transcription by RNAPII in vitro but also its coupled nucleotide excision repair (NER) activity. This strongly implicated TFIIH, the only RNAPII general factor involved in NER, as the relevant target. Subsequent biochemical experiments with purified TFIIH confirmed this and further showed that triptolide specifically binds to the XPB subunit of TFIIH and inhibits its ATPase activity. Intriguingly, triptolide did not affect the helicase activity of XPB. Nonetheless, the XPB ATPase inhibition activity of triptolide was shown to be critical for its physiological effects, as it strictly correlated with antiproliferative effects in synthesized triptolide variants. This is in agreement with the model positing that the key ATPase-dependent activity of XPB during initiation is translocation of downstream DNA into the RNAPII active site, rather than direct DNA unwinding [18].

Triptolide and some of its variants have been tested in clinical trials but have had limited success, primarily owing to their high toxicity (an expected consequence of their potent effect on the general transcription machinery). A recent attempt to circumvent this problem involved synthesis of glucose-conjugated triptolide [117]. This compound was designed to target cancer cells, given their enhanced requirement for glucose, and to improve solubility. Although glucose conjugation blocked the effects of triptolide on XPB in vitro, the modified compound retained anticancer activities against cells and in animal models. In addition, it was less cytotoxic and tolerated at higher doses than triptolide. The in vivo activity of the modified compound was likely due to its intracellular conversion into triptolide.

## 6. Compounds Targeting RNAPII

Drugs that target RNAPII itself have also been shown to be of therapeutic benefit in certain types of cancers. Actinomycin D (ActD) is commonly used as an experimental tool to inhibit transcription. It was originally developed as an antibiotic and was the first drug of this class to be shown to have therapeutic benefit in cancer. ActD is still used clinically in the treatment of Wilm’s tumor, rhabdomyosarcoma, and Ewing’s sarcoma [118]. ActD is one of several DNA intercalating agents among cancer chemotherapy drugs. These inhibit transcription by causing changes in DNA topology. As such, these agents act broadly to affect transcription by all eukaryotic RNA polymerases. ActD is in fact most effective in blocking RNAPI transcription. A preferential effect on RNAPI transcription has also been noted for CX-5461 and BMH-21, two more recently described compounds with promising anticancer properties that intercalate into double stranded DNA [119]. Preference for RNAPI may arise from the affinity of these compounds for G-quadruplex structures present in GC-rich ribosomal DNA repeats that are transcribed by RNAPI [119]. Global inhibition of either RNAPI or RNAPII may, thus, be an effective strategy for arresting cancer cell growth.

Alpha-amanitin (α-AMA) is a fungal toxin that selectively inhibits RNAPII-mediated transcription. It is known that α-AMA directly interferes with the mobile elements in the RNAPII catalytic center, the bridge helix, and the trigger loop, and is thought to prevent RNAPII translocation after nucleotide addition [34,120]. Co-crystal structures of RNAPII with bound α-AMA reveal that α-AMA binding induces a novel conformation of the trigger loop that is trapped in a “hybrid” state between open and closed. The 3′ end of the RNA is localized to the site of nucleotide addition, preventing entry of the next NTP substrate and RNAPII translocation [37].

Systemic administration of α-AMA is, unsurprisingly, extremely toxic, but genomic and proteomic studies have indicated patient populations where targeted use of α-AMA or its use as part of combination therapy could be rationalized. One study focused on drug-tolerant cells (DTCs) isolated from cancer cell lines treated with standard chemotherapeutic agents (cisplatin, docetaxel) or targeted agents (gefitinib, sorafinib) [121]. The emergence of DTCs in vitro was strongly inhibited by α-AMA treatment. Peritoneal injection of DTCs into nude mice formed highly aggressive malignancies (peritonitis carcinomatosa; PC), however co-administration of cisplatin and a non-toxic dose of α-AMA (0.4 mg/kg) blocked PC formation. This suggests that global RNAPII inhibition could be an effective way to counter transcriptional changes that lead to malignancy.

A second study revealed that many cancers might be vulnerable to treatment with RNAPII inhibitors due to hemizygous loss of the *POLR2A* gene encoding RPB1 [122]. *POLR2A* is in close proximity to *TP53* (encoding the tumor suppressor p53) on chromosome 17, and genomic deletions that remove *TP53* in colorectal tumors frequently remove neighbouring genes, including *POLR2A*. Hemizygous loss of both *TP53* and *POLR2A* was observed in some colorectal cancer (CRC) cell lines, which correlated with a reduced expression of RPB1 and a markedly increased sensitivity to α-AMA in vitro. These effects were demonstrated to be specifically due to the loss of *POLR2A* using engineered CRC cell lines. The α-AMA treatment sensitized these cells to treatment with standard chemotherapy agents used in CRC treatment, such as 5-fluorouracil, oxaliplatin, and 7-ethyl-10-hydroxy-camptothecin. These findings also translated into a potential therapeutic strategy in animal models. To avoid the toxicity associated with systemic α-AMA administration, α-AMA was covalently coupled to a monoclonal antibody against EpCam, an antigen overexpressed in adenocarcinoma. Mice xenografted with cell lines that were *POLR2A*+/- developed colorectal tumors, however tumor growth was significantly suppressed by i.p. injection of antibody-coupled α-AMA. A similar effect could be achieved by administration of *POLR2A*-directed siRNAs packaged in nanoliposomes [122]. These treatments were not associated with liver or kidney toxicity caused by systemic α-AMA administration, highlighting their potential therapeutic value. Given that hemizygous somatic loss of *TP53* is a feature of many cancers, targeted RNAPII inhibition may be a generally applicable therapeutic approach in cancer.

Somatic missense mutations in *POLR2A* have been identified in a subset of benign meningiomas, common intracranial tumors that arise from the membranes surrounding the brain and spinal cord [123]. *POLR2A* mutant tumors are phenotypically distinct from other characterized meningiomas and lack any of the known driver mutations associated with these tumors (which are found in genes encoding transcriptional regulators SMARCB1, TRAF7, NF1, and KLF4). This suggests that the mutations affect cell growth and division through a unique mechanism. The mutations cluster in the highly conserved “dock” domain of RNAPII, which interacts with TFIIB and is necessary for positioning of RNAPII with respect to TFIID and the correct transcription initiation site downstream of the core promoter. Interestingly, there was no evidence for aberrant mRNA start sites in transcriptome profiling experiments, and the mutations were instead found to misregulate specific genes involved in meningeal cell differentiation. More detailed study of transcription and mRNA processing at individual gene sites is needed to understand the mechanistic basis for these effects, however they further suggest that it is possible to identify subsets of individuals that may benefit from targeted manipulation of RNAPII activity.

## 7. Concluding Remarks

Targeting general transcriptional mechanisms in disease is a novel therapeutic approach that still requires validation in clinical settings. Nonetheless, initial indications are promising, and further tests of these agents in combination with other targeted or traditional therapies is likely to expand the potential clinical utility. Here, we have focused primarily on studies in cancers, however comparable states of transcriptional addiction are present in other disease states [2]. As more data become available on the behavior of these agents in humans, the key challenge of targeting specific cells or tissues will need to be addressed. This will likely be a limiting factor in the development of transcription-targeted drugs into broadly applicable therapeutics.

## Figures and Tables

**Table 1:** List of transcriptional inhibitors with target specificities, chemical structures, and current clinical trials. Available target specificities are listed as described in the current literature. Current clinical trials are those presently listed on clinicaltrials.gov with “recruiting”, “enrolling by invitation”, and “active, not recruiting” statuses.

**Pan-CDK Inhibitors**			
**Inhibitor Name**	**Target Specificity**	**Compound Structure**	**Listed Clinical Trials**
Flavopiridol (Alvocidib)	CDK1 - IC_50_ 30 nM [41] CDK2 - IC_50_ 170 nM [42] CDK4 - IC_50_ 100 nM [41] CDK7 - IC_50_ 875 nM [42] CDK9 - IC_50_ 20 nM [42]	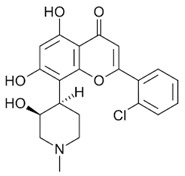	Three phase I trials ongoing for acute myeloid leukemia in combination with venetoclax and decitabine
Roscovitine (Seleciclib)	CDK1 - IC_50_ 650 nM [43] CDK2 - IC_50_ 700 nM [43] CDK5 - IC_50_ 160 nM [43] CDK7 - IC_50_ 460 nM [43] CDK9 - IC_50_ 600 nM [44]	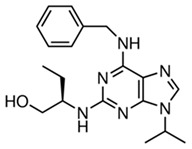	Single-agent phase II trial for Cushing’s disease
SNS-032	CDK2 - IC_50_ 48 nM [45] CDK7 - IC_50_ 62 nM [45] CDK9 - IC_50_ 4 nM [45]	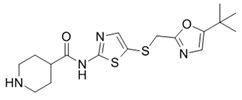	None currently ongoing
Dinaciclib	CDK1 - IC_50_ 3 nM [46] CDK2 - IC_50_ 1 nM [46] CDK5 - IC_50_ 5 nM [46] CDK9 - IC_50_ 9 nM [46]	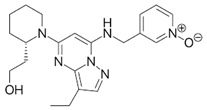	Four phase I combination (venetoclax, pembrolizumab, veliparib) and single-agent trials and one phase II trial for melanoma
AT7519M	CDK1 - IC_50_ 210 nM [38] CDK2 - IC_50_ 47 nM [38] CDK4 - IC_50_ 100 nM [38] CDK5 - IC_50_ 13 nM [38] CDK6 - IC_50_ 170 nM [38] CDK9 - IC_50_ <10 nM [38]	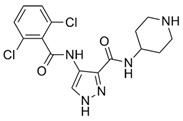	One phase I trial in combination with onalespib for solid tumors
TG02	CDK1 - IC_50_ 9 nM [39] CDK2 - IC_50_ 5 nM [39] CDK3 - IC_50_ 8 nM [39] CDK5 - IC_50_ 4 nM [39] CDK9 - IC_50_ 3 nM [39]	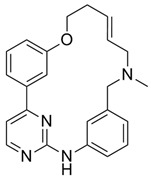	Three phase I studies for glioblastoma in combination with temozolomide (TMZ), and one for glioma in combination with pembrolizumab and TMZ
**CDK7**			
**Inhibitor Name**	**Target Specificity**	**Compound Structure**	**Listed Clinical Trials**
THZ1	CDK7 - IC_50_ 3.2 nM [47] CDK12 - IC_50_ 250 nM [47]	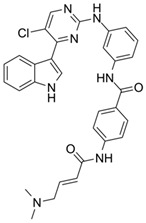	None currently ongoing
SY-1365	CDK2 - IC_50_ 2600 nM CDK7 - IC_50_ 20 nM CDK9 - IC_50_ 670 nM	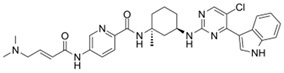	Single-agent phase I for advanced solid tumors
BS-181	CDK2 - IC_50_ 880 nM [48] CDK7 - IC_50_ 20 nM [48] others - >1000 nM [48]	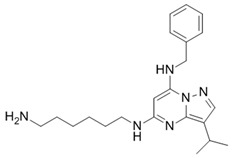	None currently ongoing
ICEC0942 (CT7001)	CDK2 - IC_50_ 620 nM [49] CDK7 - IC_50_ 4 nM [49] CDK9 - IC_50_ 1200 nM [49] others - >1000 nM [49]	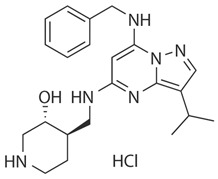	Phase I/II for advanced malignancies
**Mediator Kinase**			
**Inhibitor Name**	**Target Specificity**	**Compound Structure**	**Listed Clinical Trials**
Cortistatin A	Cdk8 - IC_50_ 12 nM Cdk8 - K_d_ 17 nM Cdk19 - K_d_ 10 nM	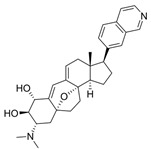	None currently ongoing
CCT251545	CDK8 - IC_50_ 5 nM [48] CDK19 - IC_50_ 6 nM [48]	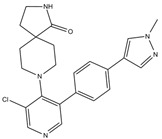	None currently ongoing
SEL120-34A	CDK8 - IC_50_ 4.4 nM [49] CDK19 - IC_50_ 10.4 nM [49]	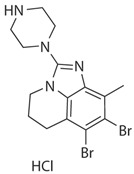	None currently ongoing
**CDK9**			
**Inhibitor Name**	**Target Specificity**	**Compound Structure**	**Listed Clinical Trials**
BAY1143572 (Atuveciclib)	CDK9 - IC_50_ 13 nM [50]	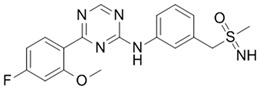	None currently ongoing
BAY1251152	CDK9 - IC_50_ 3 nM	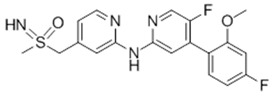	Single-agent phase I trial for advanced solid neoplasms
**CDK12 and CDK13**			
**Inhibitor Name**	**Target Specificity**	**Compound Structure**	**Listed Clinical Trials**
THZ531	CDK12 - IC_50_ 158 nM [29] CDK13 - IC_50_ 69 nM [29]	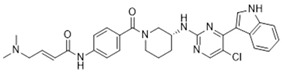	None currently ongoing
**TFIIH subunit XPB**			
**Inhibitor Name**	**Target Specificity**	**Compound Structure**	**Listed Clinical Trials**
Triptolide	XPB ATPase - IC_50_ 145 nM [51]	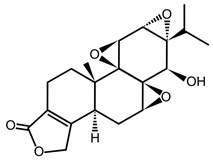	Phase I trials for advanced solid tumors in combination with paclitaxel and for HIV; phase II trial for refractory pancreatic cancer
**RNA Polymerase**			
**Inhibitor Name**	**Target Specificity**	**Compound Structure**	**Listed Clinical Trials**
Actinomycin D	RNAPI > RNAPII > RNAPIII [52]	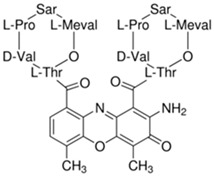	Four phase II and six phase III trials as single-agent or combination therapies for various cancers
α-amanitin	RNAPII [52]	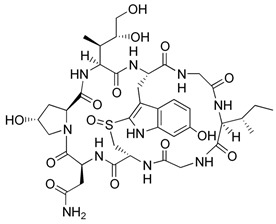	None currently ongoing
